# Acupoint catgut embedding for the treatment of obesity in adults: A systematic review protocol

**DOI:** 10.1097/MD.0000000000014610

**Published:** 2019-02-22

**Authors:** Xianming Wu, Qian Mo, Ting He, Na Zhi, Yu Huang, Shuo Yang

**Affiliations:** a Guiyang University of Chinese Medicine; b Department of Acupuncture, the Second Affiliated Hospital of Guiyang University of Chinese Medicine, Guiyang, China.

**Keywords:** acupoint catgut embedding, obesity, protocol, systematic review, weight

## Abstract

**Background:**

Obesity is the biggest chronic health problems among adults worldwide and the main predisposing factor in many types of systemic diseases such as hypertension, diabetes, and so on. In clinical reports on Traditional Chinese Medicine, acupoint catgut embedding has been shown to improve various clinical indicators for diseases including obesity and body mass index (BMI), but the safety of this and method has not been assessed.

**Methods:**

This systematic review searched the following 8 databases between from January 2015 to December 2018: the Cochrane Central Register of Controlled Trials, PubMed, EMBASE, the China National Knowledge Infrastructure, the Chinese Scientific Journal Database, the Wan-fang Database, the China Doctoral Dissertations Full-text Database and the China Master's Theses Full-text Database, and will manually searched the list of medical journals as a supplement. RCTs containing acupoint catgut embedding method for the treatment of obesity will be included. By reading the titles, abstracts and full texts, the 2 reviewers will independently complete the studies selection, data extraction, and quality assessment. The bias risk assessment, data synthesis, and subgroup analysis were performed using Revman 5.1 software.

**Results:**

The primary outcome measures include weight, improvement rate, secondary outcome measures include BMI, waist circumference, hip circumference, waist-to-hip ratio, fat percentage, and so on. The safety assessment includes the incidence of adverse events. The results will be displayed as the risk ratio of the dichotomous data, the standardized mean difference or weighted mean difference for the continuous data.

**Conclusion:**

This systematic review will retrieve clinical randomized controlled trials (RCT) on acupoint catgut embedding for obesity in 8 databases, aiming to describe and update existing evidence on the efficacy and safety of acupoint catgut embedding for obesity in adults.

**PROSPERO registration number:**

CRD42018098793.

**Key Points**

- This systematic review will assess the efficacy and safety of acupoint catgut embedding method in the treatment of obesity and will provide high-quality, current evidence for patients and clinicians seeking safe and effective treatments.
- The authenticity of the studies included in this systematic review will be confirmed by searching for published protocols, contacting the first author or correspondent author, and the 2 reviewers will independently complete study selection, data extraction, and quality assessment.
- Due to the variety of acupoint catgut embedding methods for the treatment of this disease and inherent, data heterogeneity, the difficulty of data synthesis and subgroup analysis is increased.
- Due to the language barriers (only Chinese and English studies will be read),
- Incompleteness of databases or other medical journal searches, some related studies may be missed, so the ability of this study to produce high-confidence conclusions might be limited.

## 1 Introduction

The International Obesity Task Force defines adult obesity as a chronic metabolic disease of those adults with a body mass index (BMI) over 30 kg/m^2^.^[1,2]^ With the improvement of living standards and lifestyle changes, the proportion of obese people globally has increased year by year. Obesity not only affects the appearance, but also is a highly predisposing factor for a range of diseases (hypertension, type II diabetes, coronary heart disease, and breast cancer). Obesity is a decreased quality of life and damage to physical and mental health is the biggest chronic health problem among adults worldwide and has become one of the main causes of disability or death.^[3–5]^ One study predicts that by 2030, there may be 2.2 billion overweight and 1.1 billion obese people in the world.^[6]^

Western medicine mainly adopts dietetic regulation, exercise therapy, behavioral therapy, drug therapy, surgical treatment to address obesity.^[7]^ Among them, dietetic regulation, exercise therapy, and behavioral therapy have good short-term effects, but the clinical effect varies greatly, mainly depending on patient compliance.^[8]^ Additionally, adverse reactions to drug treatment involving the nervous, cardiovascular and other systems (dizziness, anxiety, nausea, and vomiting, etc), surgical treatment is easy to cause postoperative complications, clinical application restrictions.^[9,10]^

Acupuncture to treat disease has a history of more than 2000 years in China which is based on traditional medical theory. After acupuncture needles are inserted into acupoints, corresponding methods are used to stimulate inner energy to prevent diseases or improve health.^[11–14]^ Acupoint embedding therapy refers to the use of sterile tweezers to put 3-0 catgut (1–1.5 cm) into the needle tip of No. 9 disposable sterile needles, the catgut is parallel with the inner edge of the needle tip, and the needle is followed by a blunt acupuncture needle, inserting the sterile needle into the disinfected acupuncture points. After getting deqi, the acupuncture needle is pushed in while withdrawing the sterile needle, leaving the catgut at the acupuncture point.^[15]^ Various factors are needed to achieve the treatment of diseases by regulating qi-blood and meridians, as an extension and development of acupuncture therapy. This concept combines the advantages of the 3 points of “acupoint, needle and catgut” into a comprehensive treatment method with multiple therapies and multiple effects.^[16,17]^ In addition to stimulating acupoints to coordinate zang-fu organs, supplementation of deficiencies draining repletion, regulating qi-blood, as well as acupuncture and needle retention there is also the effect of prolonging the time of acupuncture, which has its own unique advantages.^[18,19]^

In recent years, the improvement rate of acupoint catgut embedding in the treatment of adult obesity is between 76.7% and 95%.^[20–22]^ Remarkably, in databases such as Pubmed, there is only 1 relevant literature.^[23]^ Therefore, the acupoint catgut embedding treatment for obesity lacks high-quality clinical research evidence, its efficacy and safety are not clear, the existing clinical evidence needs to be identified, evaluated, graded, and summarized, providing a theoretical basis for patients and clinicians. Therefore, this systematic review will summarize the efficacy and safety of acupoint catgut embedding methods for the treatment of adult obesity. When possible, short-term and long-term outcomes will be assessed.

## 2 Criteria for including study inclusion

### 2.1.1 Types of studies

All randomized controlled trials (RCTs) on the treatment of obesity by acupoint catgut embedding, regardless of language and publication type. Exclusion non-RCTs, retrospective studies, before-and-after control studies, or studies on the mechanism of action.

### 2.1.2 Types of participants

Those diagnosed as obese, older than 18 years of age, while gender and ethnicity will not be limited. Those with secondary obesity caused by other diseases or drugs will be excluded.

### 2.1.3 Types of interventions

Interventions in the observation group included simple acupoint catgut embedding and acupoint catgut embedding combined with other therapies. The control intervention group included no active intervention, sham acupoint catgut embedding, and drugs. We will include the following comparisons:

(1) Acupoint catgut embedding is compared with other therapies (acupuncture, electroacupuncture, cupping, auricular point application, etc).
(2) Acupoint catgut embedding combined with other therapies compared with other therapies.
(3) Comparison of acupoint catgut embedding and no active intervention.
(4) Comparison of acupoint catgut embedding and sham acupoint catgut embedding.

If there were multiple intervention groups, and each intervention group will be compared to a single control group.

### 2.1.4 Types of outcomes

The primary outcomes will be: weight and improvement rate.

The secondary outcomes will be: BMI, waist circumference (WC), hip circumference (HC), adiposity, waist-to-hip ratio, and fat percentage.

Safety outcomes will be: reported incidence of all adverse events, such as sharp pain, subcutaneous hematoma, fatigue, palpitations, and so on.

### 2.2 Search methods for identification of studies

Design and implementation of a search strategy will be based on the Cochrane handbook instruction manual.^[24]^

#### 2.2.1 Electronic search

Will search the following databases (Table 1):

**Table 1:**
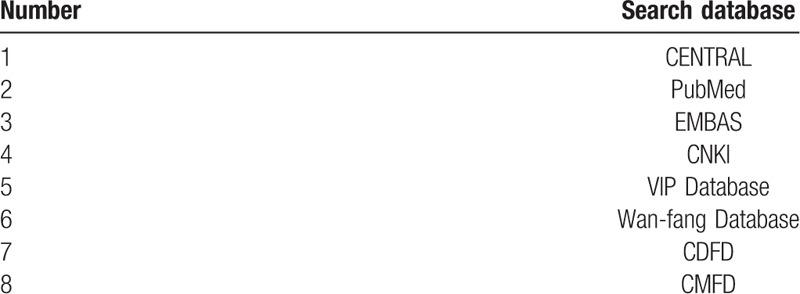
Retrieved database.

Search terms will include acupoint catgut embedding; catgut embedding; catgut implantation; obesity studies will be examined regardless of language and publication type, and will manually searched the list of medical journals as a supplement, such as: Chinese Acupuncture & Moxibustion, Acupuncture Research, Journal of Traditional Chinese Medicine. The search was completed from January 2015 to December 2018.

In addition, for studies without the full text, we will try to contact the first author or correspondent author for the full text.

### 2.3 Data collection and analysis

#### 2.3.1 Selection of studies

**Figure 1:**
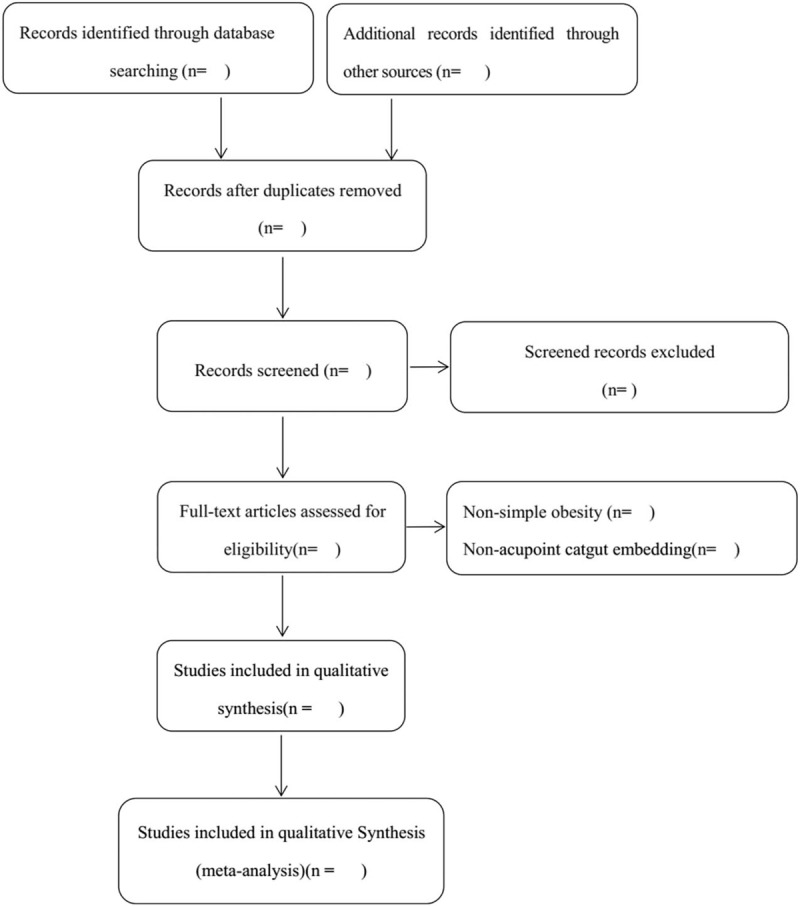
Flow chart of the trial selection process for this systematic review.

Our screening process will be discussed and developed before the selection of studies, and the results will be curated using EndNote software. According to the inclusion and exclusion criteria, appropriate studies will be identified and collected by 2 independent reviewers (XW and QM) after reading the titles, abstracts or full texts. The authenticity of this inclusion analysis will be verified by retrieving published protocols, contacting the first author or correspondent author. Any disagreements over inclusion will be decided by a third reviewer (SY) after discussion. Details of the selection process are shown in Figure 1.

#### 2.3.2 Data extraction and management

All reviewers discussed and produced a trial data extraction form based on the Cochrane Handbook. Two independent reviewers (XW and QM) extracted data from the included studies. The extracted data includes general information (author, date of publication, journal, etc), participant, randomization, blinding, allocation concealment, intervention methods, outcomes, selective reports, and other items (such as funding sources and ethical approvals). Any disagreement regarding data extraction will be resolved through discussion with the third reviewer (SY). If there is incomplete data, the published protocols will be retrieved, and the first author or corresponding author will be contacted.

#### 2.3.3 Assessment the risk of bias in studies

Two reviewers (XW and QM) will use the “bias risk” tool of the Cochrane Handbook (V.5.1.0) to independently evaluate biased risk assessment content: random sequence generation, allocation concealment, blinding, incomplete data, selective report, and other issues. Any disagreements will be resolved through discussion or negotiation with the third reviewer (SY). Biased results will be classified into “high risk of bias,” “ low risk of bias,” or “unclear risk of bias.”

#### 2.3.4 Measures of treatment effect

For dichotomous data, the results will be presented as the risk ratio of 95% confidence intervals. For continuous data, a weighted mean difference of 95% confidence intervals was used.

#### 2.3.5 Deal with missing data

For studies with missing or ambiguous data, the reviewer will contact the first author or corresponding author by phone, email, and so on. A data extraction form will be used to collect the missing data. Statistical and omission of missing data analysis will use intention-to-treat (ITT) analysis, including case analysis and complete ITT analysis.

#### 2.3.6 Assessment of heterogeneity

Heterogeneity assessment will be performed prior to meta-analysis, and the clinical and method heterogeneity will be estimated based on data recorded in the extracted form. Heterogeneity will be calculated using the Mantel–Haenszel *χ*^2^ test. *P*-value <.10 or high *I*^2^ values indicates that the heterogeneity is statistically significant. The Cochrane Handbook divides *I*^2^ values into 4 categories: 0% to 40% represents less or no heterogeneity; 30% to 60% is moderate heterogeneity; 50% to 90% means greater heterogeneity; 75% to 100% corresponds to considerable heterogeneity. The size of the impact, the direction of the outcomes and the strength of the evidence also affect heterogeneity.

#### 2.3.7 Assessment of reporting biases

All reviewers retrieve the published trial protocols, contact the first author or correspondent author of the study for information on selective reports. According to the criteria of the “Critical Risk Assessment Tool” of the Cochrane Handbook (V.5.1.0), the collected results will be judged as having a “high-risk bias,” “low-risk bias,” and “unclear risk bias.” In the subsequent meta-analysis, if more than 10 trials are included, a funnel plot will be used to evaluate potential publication bias. The asymmetry of the funnel plot may be due to publication or related bias, or it may be due to systematic differences between the various types of research. If the reason for the asymmetry is clear and the clinical diversity of the studies is to be further studied, it should be explained. Because the evolution of this graph is subjective, Egger method will also be used to evaluate report bias.

### 2.4 Data synthesis

Data synthesis will be performed using the Review Manager (V.5.2) statistical software. More than 2 clinical, methodological, and heterogeneous studies will be used to perform a meta-analysis to calculate the risk ratio of 95% confidence intervals for the dichotomous data. If the included studies are heterogeneous and have a *P*-value <.10, the risk ratio, weighted mean difference will be calculated using the random-effects model, and conversely calculated by the fixed-effect model.

### 2.5 Subgroup analysis

Subgroup analysis will be based on different types of acupoint catgut embedding therapy, treatment duration, and curative effects. The incidence of adverse events will be statistics and assessed using descriptive methods.

### 2.6 Sensitivity analysis

Sensitivity analysis will be performed if no errors occur during data input steps but subgroup analyses still have significant heterogeneity. After excluding low-quality studies, meta-analysis will be repeated and the results of the 2 meta-analyses will be compared. Case studies and complete ITT analysis can be used based on the sample size of the study, the strength of the evidence, and the influence on the pooled effect size.

## 3 Discussion

Obesity is a major predisposing factor for many types of systemic diseases like hypertension and diabetes among others.^[25]^ It can affect patient quality of life and damaging their physical and mental health. In Chinese clinical reports, acupoint catgut embedding is a safe and effective intervention for obesity. In 2015, Guo et al published a meta-analysis on acupoint catgut embedding and concluded that the effects of obesity treated by acupoint catgut embedding were superior or equal to other interventions in improving body weight, BMI, improvement rate, WC, and HC. Further high-quality studies with the rigorous designed and Food and Drug Administration approved drugs as controls are needed to evaluate the effect of acupoint catgut embedding for treating obesity.^[26]^ However, that systematic review only include studies before 2014 and did not included master theses or doctoral dissertations, and only analyzed study selection (inclusive and exclusive criteria), data sources and search methods of the included studies. In 2018, Zhang et al published a network meta-analysis on acupuncture and related therapies for obesity,^[27]^ This systematic review included the method of acupoint catgut embedding, but did not targeted analyze the effectiveness and safety of acupoint catgut embedding for obesity, and the indicators analyzed were body weight and BMI. In most of the indicators of acupoint catgut embedding for obesity, the effective rate, WC, HC, and so on are also included.

Since 2015 a large number of RCTs have been published. Specifically, there have been 87 Chinese studies on acupoint embedding for obesity, including 59 journal articles, 28 master theses and doctoral dissertations, and 1 study in PubMed. However, these studies may have used random methods, allocation concealment, poor blinding methods, and no mention of the separation of evaluators may result in the data missing and selection bias affecting the observable curative effect. Observed indicators are mostly weight, improvement rate, BMI, WC, HC, waist-to-hip ratio, fat percentage, and so on, lack of some objective laboratory indicators. Short-term curative effect is better, but follow-up records are few, the long-term curative effect remains to be further discussed, so this systematic review aims to update and improve.

To improve the transparency and credibility of clinical datum, a statement published in 2004 by the International Committee Medical of Journal Editors required all clinical trials to be registered. This can be verified by retrieving the published protocol, contacting the first author or the corresponding author. Although the importance of clinical trial registration has been widely publicized, some researchers still ignore its importance, especially in China. Therefore, it is necessary to confirm the authenticity of the included studies in order to conduct a high-quality systematic review and provide evidence for patients and clinicians.

However, this systematic review will still have limitations. The acupoint catgut embedding therapy intervention methodology has significant heterogeneity, which increases the difficulty of subgroup analysis. An array of subgroup analyses may reduce the comparability of the study, increase the complexity of the meta-analysis, and limit high-confidence conclusions. Due to language barriers, this systematic review cannot be searched in more electronic databases, and some related studies may be missed. Finally, the variety of acupoint catgut embedding methods for the treatment of this disease, the intervention method has significant heterogeneity, which increases the difficulty and complexity of subgroup analysis and may limit high confidence conclusions.

## Author contributions

The protocol manuscript was drafted by XW and QM, and revised by SY. The search strategy was formulated by all authors, XW and QM will independently screen potential studies and extract data from included studies. TH, NZ, and YH will assess the risk of bias and finish data synthesis. SY will arbitrate any disagreements and ensure that no errors occur during the review. All authors have approved the publication of the protocol.

**Data curation:** Na Zhi, Yu Huang.

**Formal analysis:** Ting He.

**Investigation:** Shuo Yang.

**Methodology:** Xianming Wu, Qian Mo, Shuo Yang.

**Project administration:** Shuo Yang.

**Writing – original draft:** Xianming Wu, Qian Mo.

**Writing – review and editing:** Xianming Wu, Qian Mo.
